# I-BET726 suppresses human skin squamous cell carcinoma cell growth in vitro and in vivo

**DOI:** 10.1038/s41419-020-2515-z

**Published:** 2020-05-05

**Authors:** Zhengjun Liu, Ping Li, Yong-qiang Yang, Shang Cai, Xiangwei Lin, Min-bin Chen, Hailei Guo

**Affiliations:** 1 Department of Burn and Plastic Surgery, The First Affiliated Hospital of Wenzhou Medical University, Wenzhou, China; 2 Department of Radiotherapy and Oncology, Affiliated Kunshan Hospital of Jiangsu University, Kunshan, China; 3 Department of Radiotherapy and Oncology, the Second Affiliated Hospital of Soochow University, Suzhou, China

**Keywords:** Targeted therapies, Targeted therapies

## Abstract

Bromodomain-containing protein 4 (BRD4) is a potential therapeutic target of skin squamous cell carcinoma (SCC). I-BET726 is a novel BRD4 inhibitor. Its potential effect in skin SCC cells was tested in the present study. We show that I-BET726 potently inhibited survival, proliferation, cell cycle progression, and migration in established (A431/SCC-9/SCC-12/SCC-13 lines) and primary human skin SCC cells. I-BET726 induced significant apoptosis activation in skin SCC cells. It was more efficient in inhibiting skin SCC cells than known BRD4 inhibitors (JQ1, CPI203, and AZD5153). I-BET726 not only downregulated BRD4-regulated proteins (c-Myc, Bcl-2, and cyclin D1), but also inhibited sphingosine kinase 1 (SphK1) and Akt signalings in SCC cells. Restoring Akt activation, by a constitutively active S473D mutant Akt1 (“caAkt1”), partially inhibited I-BET726-induced cytotoxicity in A431 cells. In vivo, I-BET726 oral administration potently inhibited A431 xenograft growth in severe combined immunodeficient mice. Downregulation of BRD4-regulated proteins and inhibition of the SphK1-Akt signaling were detected in I-BET726-treated A431 xenograft tumor tissues. Together, I-BET726 inhibits skin SCC cell growth in vitro and in vivo.

## Introduction

Skin squamous cell carcinoma (SCC) causes significant cancer-related human mortalities^1–3^. The prognosis for the advanced, metastatic, and recurrent skin SCC is poor^1–3^. Molecularly targeted therapies are desperately needed for better skin SCC therapy^1–3^.

Bromodomain and extraterminal (BET) family proteins have emerged as exciting and novel therapeutic proteins for cancer^4,5^. Bromodomain-containing protein 4 (BRD4) is the most studied and a primary member of BET family. BRD4 overexpression and/or hyperactivation is associated with tumorigenesis of hematological and solid tumors^4,5^. BRD4 regulates transcription elongation of key genes of cell proliferation, cell cycle progression, and apoptosis, including *c-Myc*, *Bcl-2*, and *cyclin D1*^5^. BRD4 inhibitors have been tested in a number of preclinical cancer studies, showing promising anticancer outcomes. Our previous study has shown that overexpression of BRD4 in human skin SCC cells can promote cell growth in vitro and in vivo^6^. BRD4 shRNA or knockout (by CRISPR/Cas9 method) potently inhibited skin SCC cell proliferation. Reversely, forced overexpression of BRD4 facilitated skin SCC cell proliferation^6^. These results suggest that targeting BRD4 could be a novel and efficient strategy against skin SCC cells.

I-BET726 (GSK1324726A) is a novel and potent inhibitor of BET family proteins, showing high affinity binding to BRD4^7^. I-BET726 competes with tetra-acetylated histones for binding to the bromodomain of BRD4^7^. I-BET726 exhibited over 1000-fold selectivity of BRD4 than the bromodomain-containing homologs^7^. The compound displayed favorable physicochemical and pharmacokinetic properties along with acceptable safety profiles, suitable for potential clinical development^7^. Its potential activity in human skin SCC cells has not been studied.

## Materials and methods

### Chemicals and reagents

I-BET726 was purchased from Adooq (Shanghai, China). MTT, AZD5153, PD98059 and LY294002, JQ1 and CPI203 were purchased from Sigma (Shanghai, China). Z-DEVD-fmk, Z-LEHD-fmk, and Z-VAD-fmk were provided by Calbiochem (La Jolla, CA). Antibodies for c-Myc (#9402), Cyclin D1 (#2922), BRD4 (#13440), Bcl-2 (#15707), SphK1 (#12071), phosphorylated (“p”)-Akt (Ser-473) (#9271), Akt1/2 (#9272), p-p44/42 MAPK (Erk1/2) (#9101) and Erk1/2 (#9102), cleaved-caspase-3 (#9664), cleaved-caspase-8 (#9496), cleaved-caspase-9 (#20750), cleaved-poly (ADP-ribose) polymerase (PARP) (#5625), β-tubulin (#15115) and β-actin (#3700) were purchased from Cell Signaling Tech (Beverly, MA).

### Cell culture

The established skin SCC cell lines, A431, SCC-9, SCC-12, and SCC-13, were purchased from the Cell Bank of Chinese Academy of Science (Shanghai, China). The cell culture procedure was described early^6^. Cells were subjected to mycoplasma and microbial contamination examination every 3–4 months. Authentication by short tandem repeat profiling, population doubling time, and morphology were routinely confirmed as well to verify the genotype. The primary human skin SCC cells were provided by Dr. Wang^8^. Cells were derived from two written-informed consent SCC patient (“C1/C2”, with PTEN depletion and p53-null) and cultured as described previously^6^. Cultures of primary human skin keratinocytes and fibroblasts were reported previously^6,9^. The protocols of using primary human cells were conducted to accordance with the Declaration of Helsinki, with approval by the Ethics Board of Wenzhou Medical University.

### MTT assay

Cells were seeded onto 96-well plates (5 × 10^3^ cells per well). After I-BET726 treatment, cell viability was tested by MTT dye assay. MTT optical densities were measured at 550 nm using a microplate reader.

### Soft agar colony formation

Following I-BET726 treatment, A431 cells were placed onto 10-cm dishes (2×10^4^ cells per treatment)^10^. I-BET726 medium was renewed every two days. After 10 days, the number of A431 colonies was counted.

### Hoechst-33342 staining of apoptotic cells

Cells were plated onto 24-well plates (2 × 10^4^ cells per well). Following the treatments, cells were stained with Hoechst-33342 (Sigma). Nuclei with intensified Hoechst-33342 condensation or fragmentation were labeled as nuclei of apoptotic cells. The nuclei of non-apoptotic cells showed the faint delicate Hoechst-33342 staining. At least 300 cells from five random-selected views (1:100 under microscope) were included to calculate the apoptotic nuclei ratio.

### BrdU assay

Cells were seeded onto 96-well plates (5 × 10^3^ cells per well), treated with I-BET726 in the presence of BrdU (10 μm, Cell Signaling Tech). BrdU incorporation was examined by an ELISA kit (Cell Signaling Tech). BrdU enzyme-linked immunosorbent assay (ELISA) OD values at 405 nm were recorded.

### EdU (5-ethynyl-20-deoxyuridine) assay

Cells were initially seeded into six-well plates (at 100,000 cells in each well). After the applied treatment, EdU Apollo-567 assay kit (RiboBio, Guangzhou, China)^11,12^ was applied to examine and quantify cell proliferation. The nuclear EdU and DAPI staining were visualized through a fluorescent microscope. Five random views with total 800–1000 cells of each treatment were included to calculate EdU/DAPI ratio.

### In vitro cell migration assay

Skin SCC cells with the applied treatments were initially seeded on the “Transwell” upper chamber (BD Biosciences, Shanghai, China), at a density of 10,000 cells in 300 μL serum-free medium (each chamber)^13^. FBS-containing complete medium was added to the lower chamber surface. After incubation for 48 h the migrated cells on the lower surface were stained and counted. Five repeated views of each condition were included to calculate the average number of migrated cells.

### Cell cycle assay

Cells with I-BET726 treatment were fixed and stained with propidium iodide (PI, 10 μg/mL, Invitrogen) and RNase (100 μg/mL, Invitrogen). A flow cytometer (BD Biosciences, Franklin Lakes, NJ) was employed to analyze DNA content. Cell cycle distribution percentages (G0/1-, S- and G2/M-phases) were recorded.

### Caspase activity assay

The cytosolic protein extracts (30 μg per treatment) were incubated with the 7-amino-4-trifluoromethylcoumarin (AFC)-conjugated caspase (−3/−8/−9) substrates (10 μg/mL each) in the caspase assay buffer. AFC intensities were tested by Infinite 200 PRO reader at 400 nm excitation and 505 nm emission.

### Annexin V assay

In brief, following the treatments, cells were stained with Annexin V-FITC (10 μg/mL) and PI (10 μg/mL) (Sigma). Afterwards, cells were detected via fluorescence-activated cell sorting on a FACSCalibur machine (BD Biosciences). Annexin V^+/+^ cells were labeled as apoptotic cells.

### Lactate dehydrogenase (LDH) assay of cell death

In brief, following the indicated treatment, medium LDH was collected and tested by a two-step LDH detection kit (Promega, Shanghai, China), and normalized to total LDH contents.

### Western blotting assay

As described^9^, the quantified protein lysates (30 μg proteins per treatment) were separated by 10% sodium dodecyl sulphate–polyacrylamide gel electrophoresis, transferred to the PVDF blots (Biyuntian). After blocking, the blots were immunoblotted with applied primary antibodies, followed by incubation with HRP-conjugated secondary antibodies. The enhanced chemiluminescence reagents (Pierce) were added to visualize the targeted protein bands. Total gray of each band was quantified by the ImageJ software^14^.

### Sphingosine kinase 1 (SphK1) activity assay

SphK1 activity was detected by a previously described protocol^15,16^. SphK1 activities were valued as pmol/hour/g protein, normalized to the control.

### Ceramide assay

Using a previously described protocol^17^, the cellular ceramide content was analyzed, and its value was expressed as fmol by nmol of phospholipid. Ceramide content in I-BET726-treated cells was always normalized to that of untreated control cells.

### Constitutively active mutant Akt1

The purified constitutively active S473D mutant Akt1 (“caAkt1”) recombinant adenovirus and empty vectors recombinant adenovirus (Ad-GFP) were both provided by Dr. Cao at Soochow University^18^. Cells were initially seeded into six-well plates (at 100,000 cells in each well). The adenovirus was added to cultured cells for 48 h. The stable cell lines were established via selection for 4–5 passages. For virus infection, cells were cultured in polybrene-containing medium.

### BRD4 knockout (KO)

The CRISPR/Cas9 BRD4-KO plasmid (sc-400519-KO-2; Santa Cruz Biotechnology, Shanghai, China) was transfected to cultured A431 cells via the Lipofectamine 2000 protocol (Invitrogen, Shanghai, China). The transfected cells were further selected with puromycin after 4–5 passages, and two stable cell lines were established (“BRD4-KO-sL1/2”). Control cells were treated with an empty vector with Cas9 control construct (“Cas9-C”; Santa Cruz Biotechnology). BRD4 expression in stable cells was verified by western blotting.

### Xenograft assay

As described^6,9^ A431 cells (6 × 10^6^ cells in 200 μL matrigel/serum-free medium, for each mouse) were inoculated via subcutaneous (s.c.) injection to the right flanks of the severe combined immunodeficient (SCID) mice (female, 6–7 weeks old). Within 18–20 days the volume of each tumor was close to 100 mm^3^, and the A431 tumor xenografts were established. SCID mice were randomly assigned into three groups (10 mice per group). Every 5 days tumor volumes and mice body weights were recorded. All animal studies were performed according to the standards of ethical treatment and IACUC of Wenzhou Medical University. The protocols were approved by the Ethics Committee (2016-R311) of Wenzhou Medical University.

### Statistical analysis

The investigators were blinded to the group allocation during all experiments. In vitro experiments in this study were repeated at least three times. Data were presented as mean ± standard deviation (SD). Statistics were analyzed by one-way analysis of variance by SPSS software (21.0, Chicago, CA). To compare difference between two specific groups, a two tailed *t* test was applied (Excel 2007). *p* values < 0.05 were considered statistically different. All the protocols of this study were approved by Ethics Committee of Wenzhou Medical University.

## Results

### I-BET726 inhibits human skin SCC cell viability, proliferation, cell cycle progression, and migration

**Fig. 1:**
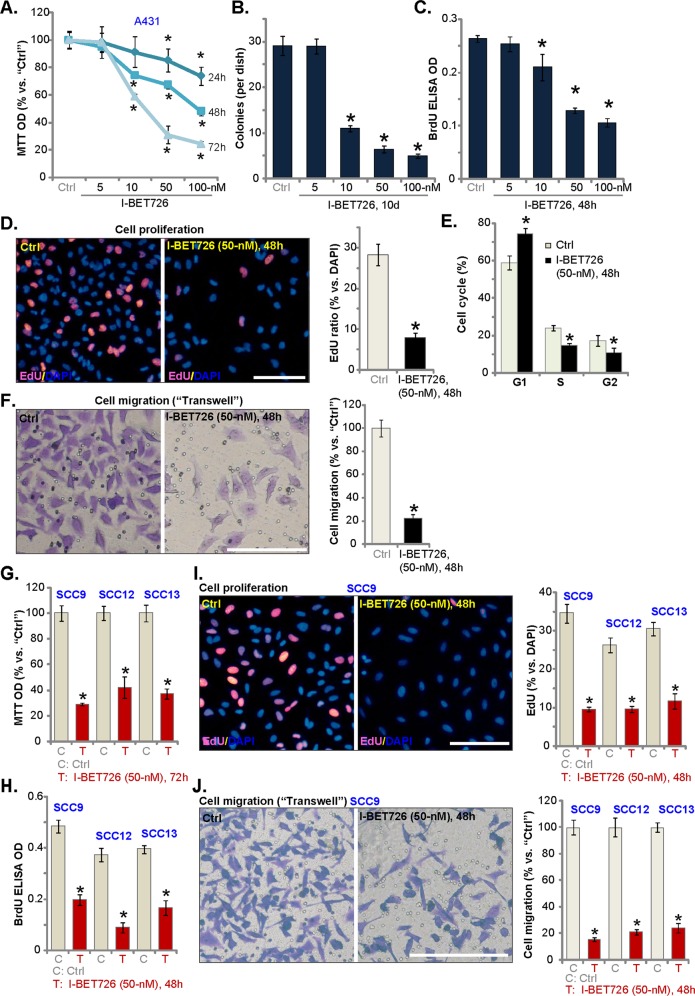
I-BET726 inhibits survival, proliferation, cell cycle progression, and migration in established SCC cells. A431 cells **a**–**f** SCC-9, SCC-12, or SCC-13 cells **g**–**j** were left untreated (“Ctrl”, same for all Figures), or treated with I-BET726 (5–100 nm), cells were further cultured in I-BET726-containing medium for indicated time periods, cell viability **a**, **g**, proliferation (**b**–**d**, **h**, **i**), cell migration **f**, **j**, and cell cycle progression **e** were tested by the appropriate assays. Data were presented as mean ± standard deviation (SD) (Same for all Figures). *n* = 5 stands for five replicate wells/dishes (Same for all Figures). *p < 0.05 vs. “Ctrl” group. All in vitro experiments in this study were repeated three times with similar results obtained. Bar = 100 μm **d**, **f**, **i** and **j**.

A431 SCC cells were treated with I-BET726 at gradually increased concentrations (5–100 nm). MTT assay results, in Fig. 1a, show that I-BET726, in a concentration-dependent manner, potently inhibited A431 cell viability. I-BET726 also displayed a time-dependent response in inhibiting A431 cell viability (Fig. 1a). The IC-50 of I-BET726 was close to 10–50 nm (72 h, Fig. 1a). A431 cell proliferation was analyzed by soft agar colony formation assay and BrdU incorporation ELISA assay. As demonstrated, I-BET726 dose-dependently decreased the number of A431 cell colonies (Fig. 1b) and BrdU ELISA OD (Fig. 1c), indicating an antiproliferative activity by I-BET726. EdU incorporation assay results, Fig. 1d, demonstrated that I-BET726 treatment (50 nm, 48 h) potently decreased EdU ratio in A431 cells, further confirming proliferation inhibition. In addition, when analyzing cell cycle progression, we show that I-BET726 (50 nm) disrupted cell cycle progression, causing G1–S arrest in A431 cells (Fig. 1e). By counting the number of the migrated cells in the “Transwell” assay, we show that I-BET726 (50 nm, 24 h) significantly inhibited A431 cell migration in vitro (Fig. 1F).

In other three established SCC cell lines, SCC-9, SCC-12, SCC-13, I-BET726 treatment (50 nm, 24–72 h) robustly decreased cell viability (Fig. 1g), BrdU ELISA OD (Fig. 1h) and EdU incorporation (Fig. 1i). Furthermore, “Transwell” assay results, Fig. 1j, demonstrated that I-BET726 inhibited in vitro migration of the tested SCC cells.

**Fig. 2:**
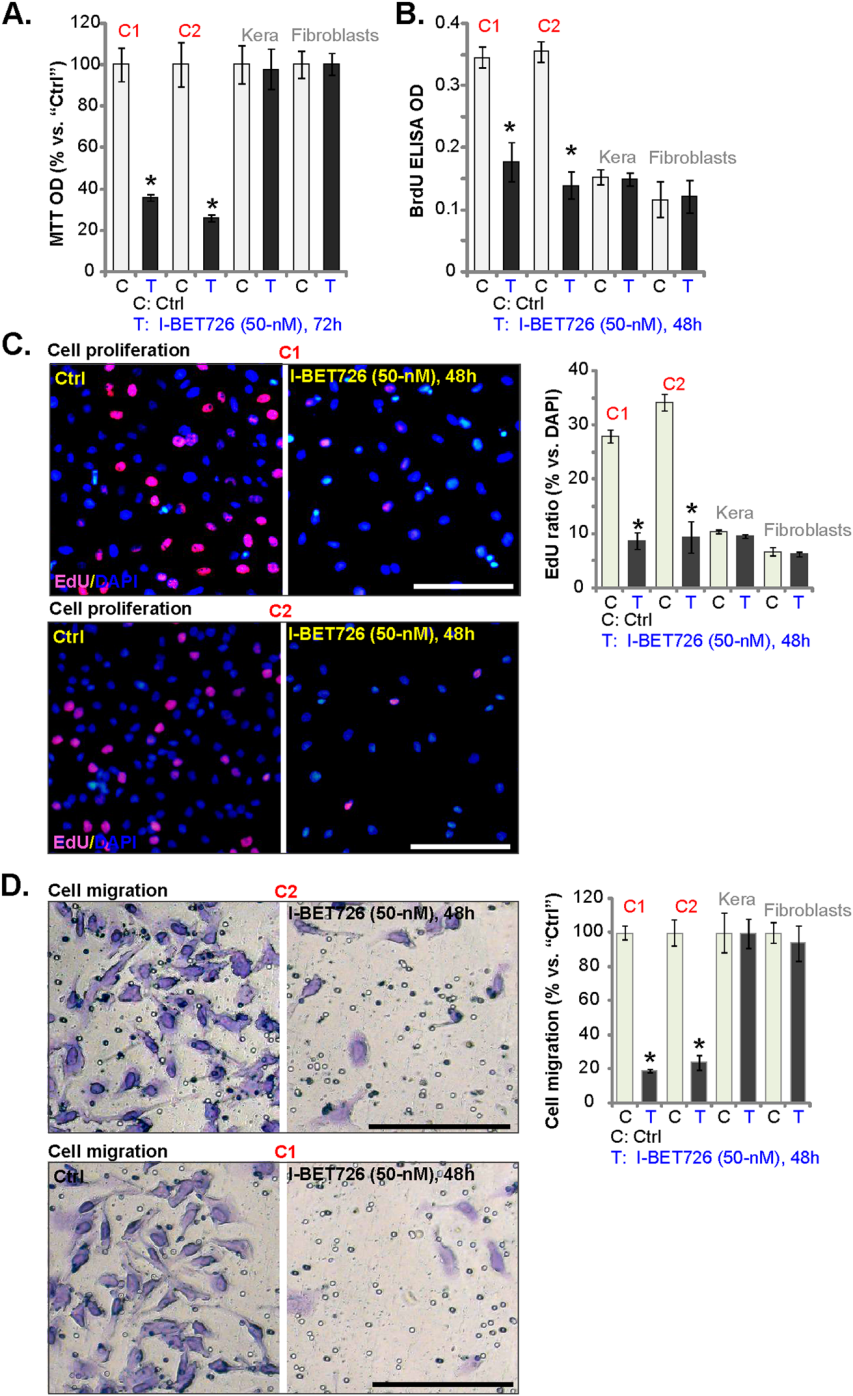
I-BET726 inhibits survival, proliferation and migration in primary human SCC cells. The primary human skin SCC cell (“C1”/“C2”), human skin keratinocytes (“Kera”) and fibroblasts were treated with I-BET726 (50 nm), cells were further cultured in I-BET726-containing medium for 48–72 h, cell viability **a**, proliferation **b**, **c**, and cell migration **d** were tested by the appropriate assays. *p < 0.05 vs. “Ctrl” group. Bar = 100 μm **c**, **d**.

Next, we tested the potential effect of I-BET726 in the primary human cells. The primary human skin SCC cells, derived from two skin SCC patients (“C1/C2”), were treated I-BET726 (50 nm). As shown, I-BET726 significantly inhibited viability (MTT OD, Fig. 2a), proliferation (BrdU ELISA OD, Fig. 2b) and EdU incorporation (Fig. 2c) of primary SCC cells. On the contrary, it was ineffective in the primary human skin keratinocytes (“Kera”) and fibroblasts (Fig. 2a–c), where BRD4 levels are low^6^. Further studies show that the BRD4 inhibitor suppressed migration of primary human SCC cells (Fig. 2d), but being invalid in skin fibroblasts and keratinocytes (Fig. 2d).

We compared the activity of I-BET726 with other known BRD4 inhibitors, including JQ1^19,20^, CPI203^6,21^, and AZD5153^22,23^. In A431 cells, I-BET726 (50 nm) was significantly more potent in inhibiting cell viability (Fig. S1A) and proliferation (Fig. S1B) than even higher concentrations of JQ1 (500 nm), CPI203 (500 nm), and AZD5153 (100 nm). These results show that I-BET726 potently inhibits survival, proliferation and migration of established and primary human skin SCC cells.

### I-BET726 induces human skin SCC cell apoptosis

**Fig. 3:**
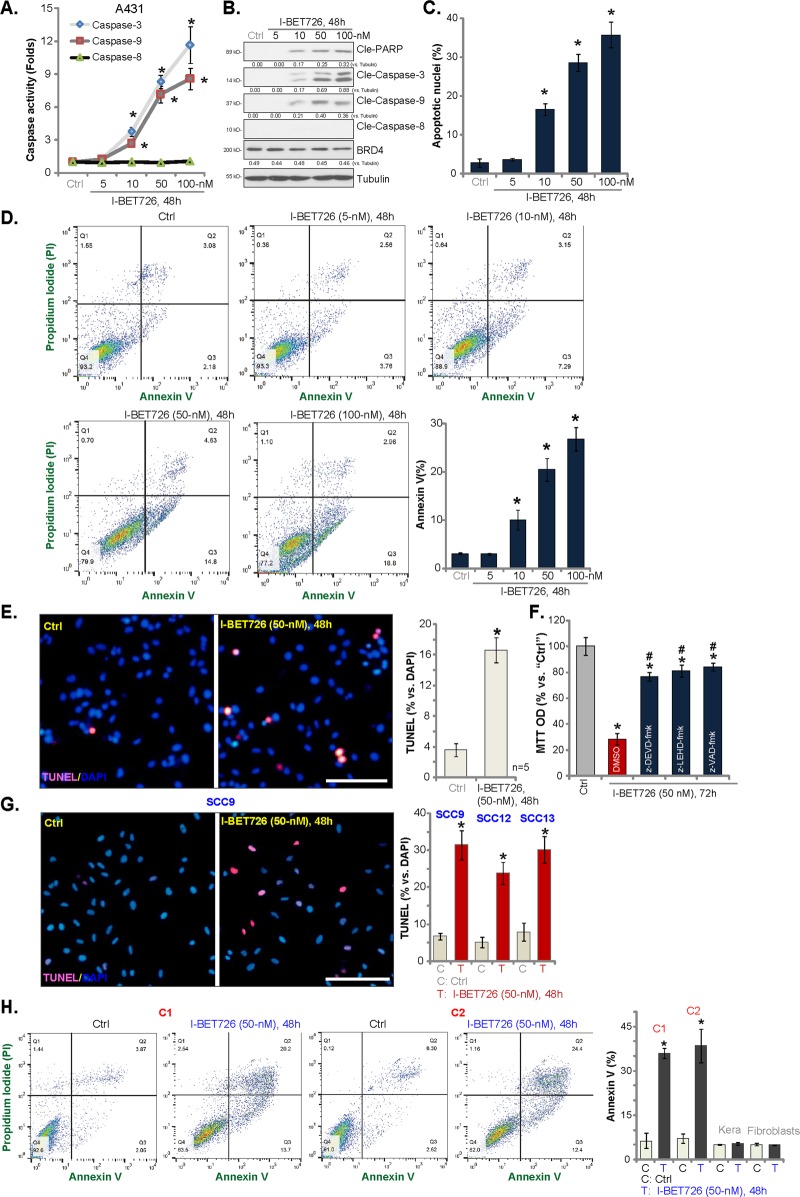
I-BET726 induces human skin SCC cell apoptosis. A431 cells **a**–**d**, SCC-9, SCC-12, or SCC-13 cells **g**, the primary human skin SCC cell (“C1”/“C2”, **h** or human skin keratinocytes (“Kera”) and fibroblasts **h** were treated with I-BET726 (5–100 nm) for 48 h, cell apoptosis was analyzed by the mentioned assays. A431 cells were pre-treated for 30 min with 50 μm of z-DEVD-fmk, z-LEHD-fmk or z-VAD-fmk, following by I-BET726 (50 nm) treatment for 72 h, cell viability was tested by MTT assay **f**. Expression of the listed proteins was quantified and normalized to loading control **b**. “DMSO” stands for vehicle control (0.1% DMSO, **f**. *p < 0.05 vs. “Ctrl” group. ^#^*p* < 0.05 vs. “DMSO” group **f**. Bar = 100 μm **e**, **g**.

Previous studies have demonstrated that BRD4 inhibition or depletion could induce apoptosis activation in human cancer cells^24–26^. The potential effect of I-BET726 on skin SCC cell apoptosis was studied next. As shown, I-BET726 dose-dependently increased the activities of caspase-3 and caspase-9 in A431 cells (Fig. 3a). Caspase-8 activity, an indicator of extrinsic apoptosis pathway activation^27^, was unaffected (Fig. 3a). Further, levels of cleaved (“Cle”)-caspase-3, cleaved-PARP and cleaved-caspase-9 were increased in I-BET726 (10–100 nm)-treated A431 cells (Fig. 3b), where cleaved-caspase-8 levels were again unaffected (Fig. 3b). BRD4 expression was unchanged by I-BET726 treatment (Fig. 3b). Following I-BET726 treatment, the percentage of nuclei with condensed/fragmented Hoechst-33342 staining (“apoptotic nuclei”) was significantly increased (Fig. 3c). Moreover, I-BET726, in a dose-dependent manner, increased the percentage of A431 cells with positive Annexin V staining (Fig. 3d). At lowest concentration (5 nm), it was ineffective on cell apoptosis (Fig. 3a–d). Therefore, I-BET726 dose-dependently induced apoptosis activation in A431 cells.

Further experimental results show that terminal deoxynucleotidyl transferase dUTP nick end labeling (TUNEL)-positive nuclei ratio was significantly increased in I-BET726 (50 nm, 48 h)-treated A431 cells (Fig. 3e). Importantly, I-BET726 (50 nm)-induced apoptosis activation was more potent than other known BRD4 inhibitors: JQ1, CPI203, and AZD5153 (Fig. S1C). Different caspase inhibitors, including the caspase-3 specific inhibitor z-DEVD-fmk, the caspase-9 specific inhibitor z-LEHD-fmk and the pan caspase inhibitor z-VAD-fmk, were utilized. In Fig. 3f, MTT assay results show that pre-treatment (for 30 min) with the caspase inhibitors significantly attenuated I-BET726 (50 nm, 72 h)-induced A431 cell viability reduction. Therefore, apoptosis activation should be the primary reason of I-BET726-induced cytotoxicity in A431 cells. In SCC-9, SCC-12, and SCC-13 cells I-BET726 treatment (50 nm, 48 h) increased TUNEL staining ratio as well (Fig. 3g).

In the primary skin SCC cells (“C1/C2”), I-BET726 (50 nm) significantly increased the Annexin V-positive cells (Fig. 3h), suggesting apoptosis activation. No significant apoptosis was yet detected in I-BET726-treated human skin keratinocytes and fibroblasts (Fig. 3h). Collectively, these results show that I-BET726 induces apoptosis activation in skin SCC cells.

### I-BET726 inhibits BRD4, SphK1, and Akt signalings in human skin SCC cells

**Fig. 4:**
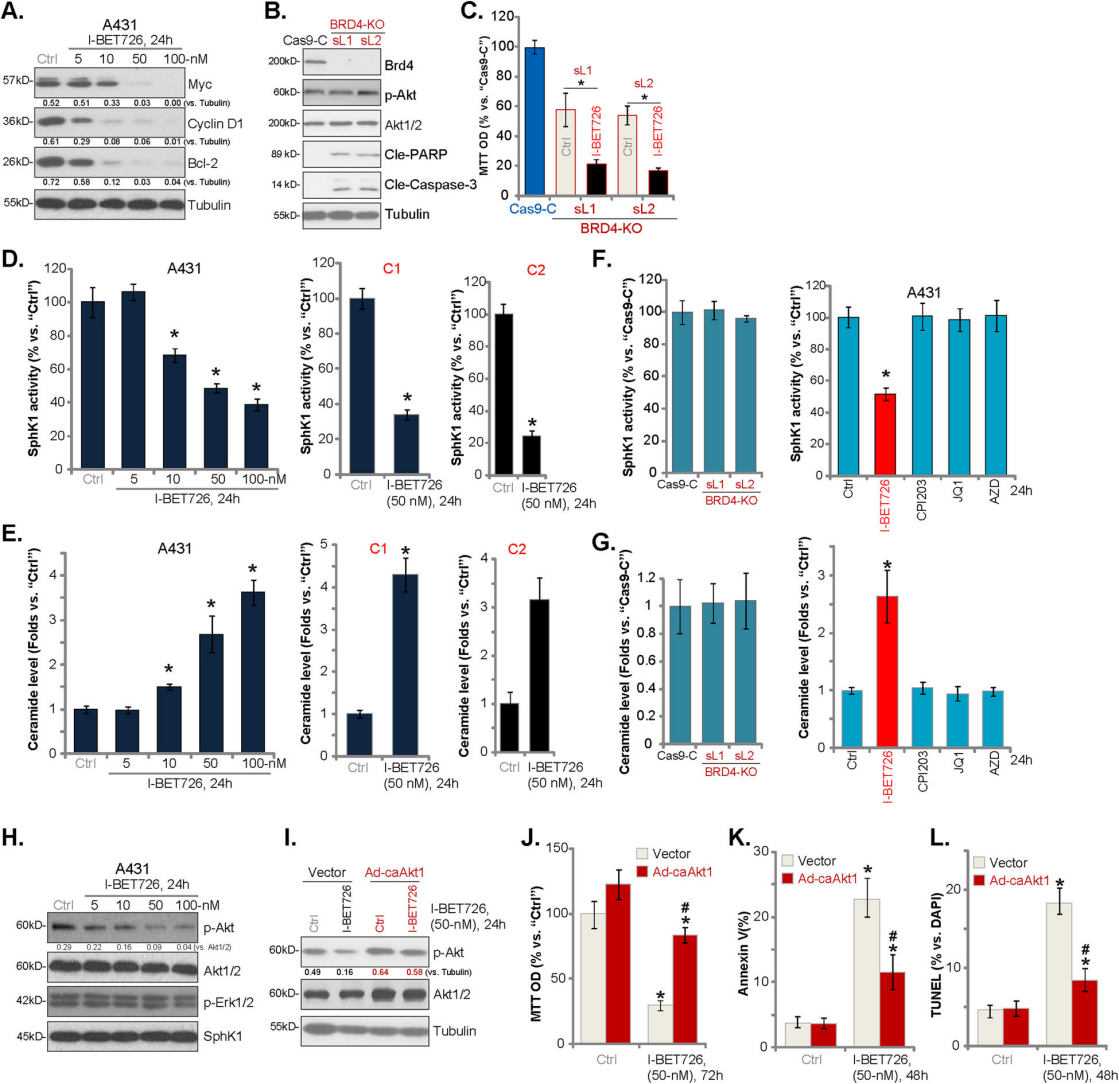
I-BET726 inhibits BRD4, SphK1, and Akt signalings in human skin SCC cells. A431 cells or the primary human skin SCC cell (“C1/C2”) were treated with I-BET726 (5–100 nm), cells were further cultured for 24 h, expression of listed proteins in total cell lysates were tested by western blotting **a**, **h**; relative SphK1 activities **d** and ceramide levels **e** were tested. Expression of BRD4 and Tubulin in the stable A431 cells with CRISPR/Cas9 BRD4-KO plasmid (“BRD4-KO-sL1/2”) or Cas9 control construct (“Cas9-C”) was shown **b**; Relative SphK1 activities (**f**, the left panel) and ceramide levels **g**, the left panel were tested. Cas9-C cells or the BRD4-KO-sL1/2 cells were treated with or without I-BET726 (50 nm) for 72 h, cell viability was tested by MTT assay **c**. A431 cells were treated with I-BET726 (50 nm), CPI203 (500 nm), JQ1 (500 nm) or AZD5153 (“AZD”, 100 nm) for 24 h, the SphK1 activities (**f**, the right panel) and ceramide levels (**g**, the right panel) were tested. Stable A431 cells with the constitutively active S473D mutant Akt1 (“caAkt1”) construct or the empty vector (“Vector”) were treated with or without I-BET726 (50 nm) for applied time periods, expression of listed proteins were shown **i**. Cell viability (MTT viability, **j** and apoptosis activation (Annexin V-FACS/TUNEL staining, **k** and **l** were tested, and results were quantified. Expression of listed proteins was quantified and normalized to the loading control (**a**, **h** and **i**). *p < 0.05 vs. “Ctrl” group. ^#^ *p* < 0.05 vs. I-BET726 treatment in “vector” control cells **j**–**l**.

BRD4 recruits and phosphorylates P-TEFb (the positive transcription elongation factor b), required for transcription elongation^4,5^. Several BRD4-dependent proteins (including c-Myc, Bcl-2, and cyclin D1) are key oncogenes^4,5^. In A431 cells, I-BET726 dose-dependently inhibited c-Myc, Bcl-2, and cyclin D1 protein expression (Fig. 4a). I-BET726 is more potent than other BRD4 inhibitors in inhibiting skin SCC cells (Figs. 1, 2), therefore it is possible that I-BET726 could regulate BRD4-indendent pathways. To support our hypothesis, the CRISPR/Cas9 BRD4-KO plasmid was transfected to A431 cells. Following selection two stable cell lines, BRD4-KO-sL1/2, were established, showing completely depleted BRD4 expression (Fig. 4b). Caspase-3 and PARP cleavages were detected in BRD4-KO cells, indicating apoptosis activation (Fig. 4b). Importantly, in the BRD4-KO A431 cells, I-BET726 (50 nm) can still induce significant cytotoxicity (MTT OD reduction, Fig. 4c), indicating the involvement of BRD4-independnent mechanism by this compound.

Existing studies have demonstrated a pivotal role of SphK1 in skin SCC progression^28,29^. SphK1 overexpression/hyperactivation promotes skin SCC cell proliferation and metastasis^28,29^. On the contrary, SphK1 inhibition or silencing shall lead to ceramide production and cell apoptosis^28,29^. SphK1 activity was therefore analyzed. We show that I-BET726 dose-dependently decreased SphK1 activity in A431 cells (Fig. 4d, left panel). SphK1 activity was inhibited by I-BET726 (50 nm) in the primary skin SCC cells (“C1/C2”) as well (Fig. 4d, right panel). Correspondingly, the cellular ceramide levels were increased in I-BET726-treated skin SCC cells (Fig. 4e). In three other established SCC cell lines, SCC-9, SCC-12, SCC-13, I-BET726 treatment (50 nm, 24 h) also induced ceramide accumulation (Fig. S2A).

Interestingly, CRISPR/Cas9-induced BRD4-KO failed to affect SphK1 activation and ceramide contents (Fig. 4f, g, the left panels). Treatment with other BRD4 inhibitors (JQ1, CPI203, and AZD5153) had no significant effect on SphK1 activation (Fig. 4f, the right panel) and ceramide production (Fig. 4g, the right panel) in A431 cells. These results suggest that SphK1 inhibition should be an unique action by I-BET726, independent of BRD4 inhibition.

Following SphK1 inhibition, accumulated ceramide could activate serine/threonine phosphatases (i.e., PP2A and PP1A) to de-phosphorylate and in-activate Akt^30–32^. In the present study, we show that Akt phosphorylation was inhibited by I-BET726 in A431 cells (Fig. 4h). Erk1/2 phosphorylation as well as SphK1 expression were not affected (Fig. 4h).

To test the link between Akt inhibition and I-BET726-induced anti-A431 cell activity, the adenovirus-packed constitutively active S473D mutant Akt1 (caAkt1, from Dr. Cao^18^) was transduced to A431 cells, which completely restored Akt activation in I-BET726-treated cells (Fig. 4i). Importantly, in A431 cells caAkt1 largely attenuated I-BET726-induced viability reduction (Fig. 4j), and apoptosis activation (Fig. 4k, l). Therefore, Akt inhibition is involved in I-BET726-induced cytotoxicity in A431 cells.

Importantly, we show that Erk inhibition by PD98059 potentiated I-BET726-induced viability reduction (Fig. S2B) and cell death (Fig. S2C) in A549 cells. Furthermore, I-BET726 inhibited, but not blocked, Akt activation in SCC cells (Fig. 4h). LY294002, the PI3K-Akt blocker^33^, intensified I-BET726-induced cytotoxicity in A431 cells (Fig. S2b, c). These results suggest that Erk and PI3K-Akt activation might be the resistance mechanisms of I-BET726 in SCC cells. Collectively, our results suggest that I-BET726 inhibits BRD4, SphK1, and Akt signalings in skin SCC cells.

### I-BET726 oral administration inhibits growth of subcutaneous A431 xenografts in SCID mice

**Fig. 5:**
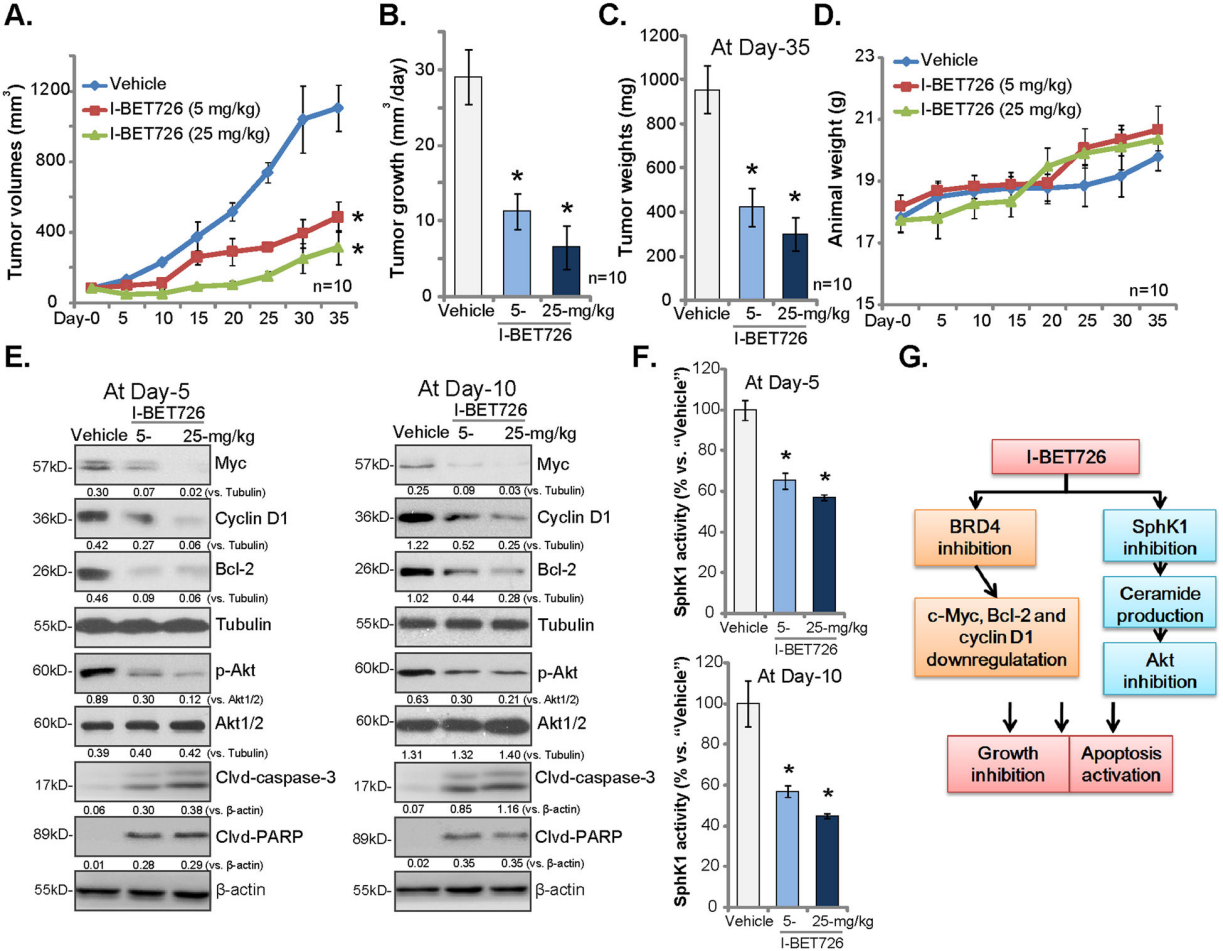
I-BET726 oral administration inhibits growth of subcutaneous A431 xenografts in SCID mice. SCID mice (10 mice per group, *n* = 10) with A431 xenografts were orally administrated with I-BET726 (gavage, 5–25 mg/kg body weight, daily for 20 consecutive days) or vehicle control (saline, “vehicle”), tumor volumes **a** and mice body weights **d** were recorded. The estimated daily tumor growth (in mm^3^ per day) was calculated using the described method **b**. At “Day-35”, tumors were individually weighted **c**. One tumor of each group was isolated at treatment “Day-5” and “Day-10”, and tumor lysates were tested by western blotting for listed proteins **e**. Relative SphK1 activities were analyzed as well (**f**, *n* = 5). The proposed signaling pathway of this study **g**. Expression of listed proteins was quantified and normalized to the loading control **e**. *p < 0.05 vs. “vehicle” group.

In order to test the activity of I-BET726 in vivo, a xenograft model was established by s.c. injection of A431 cells to the flanks of SCID mice. A431 xenograft tumors were established (close to 100 mm^3^) within 18–21 days, when I-BET726 administration was started (“Day-0”). By recording tumor volumes, we show that oral administration of I-BET726 (5–25 mg/kg body weight, gavage, daily for 20 days) potently inhibited A431 xenograft growth in SCID mice (Fig. 5a). The estimated daily tumor growth, calculated by (tumor volume at Day-35 subtracting tumor volume at Day-0)/35, was significant inhibited by I-BET726 administration (Fig. 5b). At Day-35, all tumors were individually weighted, and results show that I-BET726-treated tumors were significantly lighter than the vehicle control tumors (Fig. 5c). The animal body weights were not significantly different between three groups (Fig. 5d). Therefore, I-BET726 oral administration potently inhibited growth of subcutaneous A431 xenografts in SCID mice.

At treatment “Day-5” and “Day-10”, one tumor of each group was isolated. Analyzing tumor tissue lysates by western blots demonstrated that BRD4-regulate proteins (Bcl-2, Myc, cyclin D1) were significantly downregulated in I-BET726-treated A431 tumor tissues (Fig. 5e). As compared with vehicle control tumors, Akt inactivation (Fig. 5e) and SphK1 inhibition (Fig. 5f) were also detected in I-BET726-treated tumor tissues. The levels of cleaved-caspase-3 and cleaved-PARP were increased, indicating apoptosis activation in I-BET726-treated A431 tumor tissues (Fig. 5e). Therefore, in line with in vitro findings, I-BET726 oral administration inhibited BRD4, SphK1, and Akt signalings in A431 xenografts.

## Discussion

BRD4 binds to the chromatin to re-activate silenced genes in the process of mitosis^5,34^. In addition, BRD4 phosphorylates P-TEFb and RNA polymerase II to promote transcription elongation^5,34^. BRD4 is essential for the transcription of several key oncogenes, including *c-Myc*, *Bcl-2* and *cyclin D1*^4,35^. Moreover, BRD4 is important for the activation of oncogenic nuclear factor-kappa B signaling in cancer cells^4^. Our previous study has shown that BRD4 is overexpressed in skin SCC cells, functioning as a potential key pro-cancerous molecule^6^. Targeting BRD4, i.e., by AZD5153, can potently inhibit skin SCC cell growth, in vitro and in vivo^6^.

In the present study, we show that I-BET726, a novel BRD4 inhibitor^7^, inhibited survival, proliferation, cell cycle progression, and migration in multiple established skin SCC cell lines (A431/SCC-9/SCC-12/SCC-13) and primary human skin SCC cells. I-BET726 provoked apoptosis in skin SCC cells. It was highly potent in killing skin SCC cells, more efficient than the other known BRD4 inhibitors (JQ1, CPI203, and AZD5153). Significantly, it was non-cytotoxic to normal skin keratinocytes and fibroblasts, where BRD4 levels are extremely low^6^. In vivo, I-BET726 oral administration inhibited A431 xenograft growth in SCID mice. Downregulation of BRD4-dependent oncogenic proteins (c-Myc, Bcl-2, and cyclin D1) was detected in I-BET726-treated skin SCC cells and A431 xenografts. These results suggest that I-BET726 potently inhibited skin SCC cell progression in vitro and in vivo.

The outcomes for the current treatments of advanced skin SCC have been disappointing. The better skin SCC therapies should include rational inhibition of key molecular targets in multiple pro-survival/growth signalings. The facts that I-BET726 is more efficient than other known BRD4 inhibitors and it could still induce cytotoxicity in BRD4-KO A431 cells suggest the existence of BRD4-independent mechanisms by this compound. SphK1 promotes cancer cell viability, proliferation, and apoptosis resistance, as well as metastasis, and angiogenesis^36,37^. Previous studies have demonstrated that SphK1 is overexpressed in skin SCC, represents as a novel prognostic marker and potential therapeutic target^28,29^.

The novel findings of the study are that in skin SCC cells I-BET726 can significantly inhibit SphK1 activation, followed by pro-apoptotic ceramide accumulation and Akt inactivation. These actions by I-BET726 were independent of BRD4 inhibition. As other BRD4 inhibitors (JQ1, CPI203, and AZD5153) failed to change SphK1 activity nor Akt signaling. Concurrent inhibition of BRD4, SphK1, and Akt signalings by I-BET726 could probably explain its superior anti-skin SCC activity, better than the known BRD4 inhibitors. SphK1-Akt inhibition was also detected in I-BET726-treated A431 xenograft tissues. Thus, I-BET726 concurrently inhibits BRD4, SphK1, and Akt signalings in skin SCC cells (see the proposed signaling pathway of this study, Fig. 5g).

The effects of inhibiting Akt-mTOR in combination with other cascades pathways are being tested in a number of phase I–II clinical trials^38,39^. These trials highlighted the importance of concurrent targeting multiple oncogenic signalings to efficiently inhibit cancer cell growth and induce apoptosis^38,39^. In the current study we show that I-BET726 concurrently inhibited BRD4, SphK1, and Akt signalings in established and primary skin SCC cells. This might be one reason of its superior anti-SCC cell activity. Importantly, we show that Erk inhibition or PI3K-Akt blockage potentiated I-BET726-induced cytotoxicity in SCC cells, suggesting that Erk and PI3K-Akt activation could be key resistance mechanisms of I-BET726 in SCC cells. Although additional studies will be needed to further support this notion.

## Conclusion

I-BET726 potently inhibits BRD4, SphK1-Akt signalings, and human skin SCC cell growth, in vitro and in vivo.

## Supplementary information

Supplementary Figure 1

Supplementary Figure 2

Supplementary Figure Legends
