# Supplementary figures and images for: I-BET726 suppresses human skin squamous cell carcinoma cell growth in vitro and in vivo
Source: Cell Death Dis. 2020 May 5;11(5):318. doi: 10.1038/s41419-020-2515-z (PMC7200671; doi:10.1038/s41419-020-2515-z)

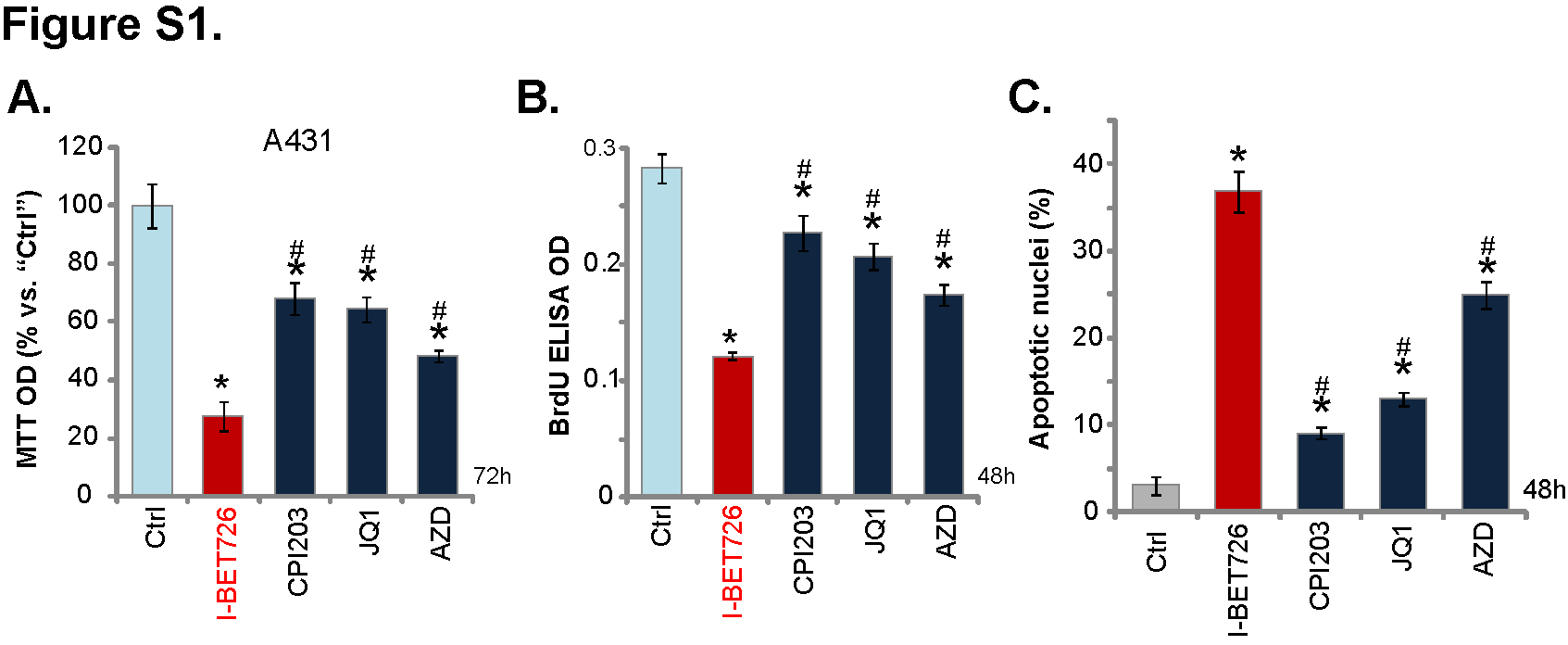

Supplement: Supplementary file 1 — Supplementary Figure 1 [file 41419_2020_2515_MOESM1_ESM.tif]

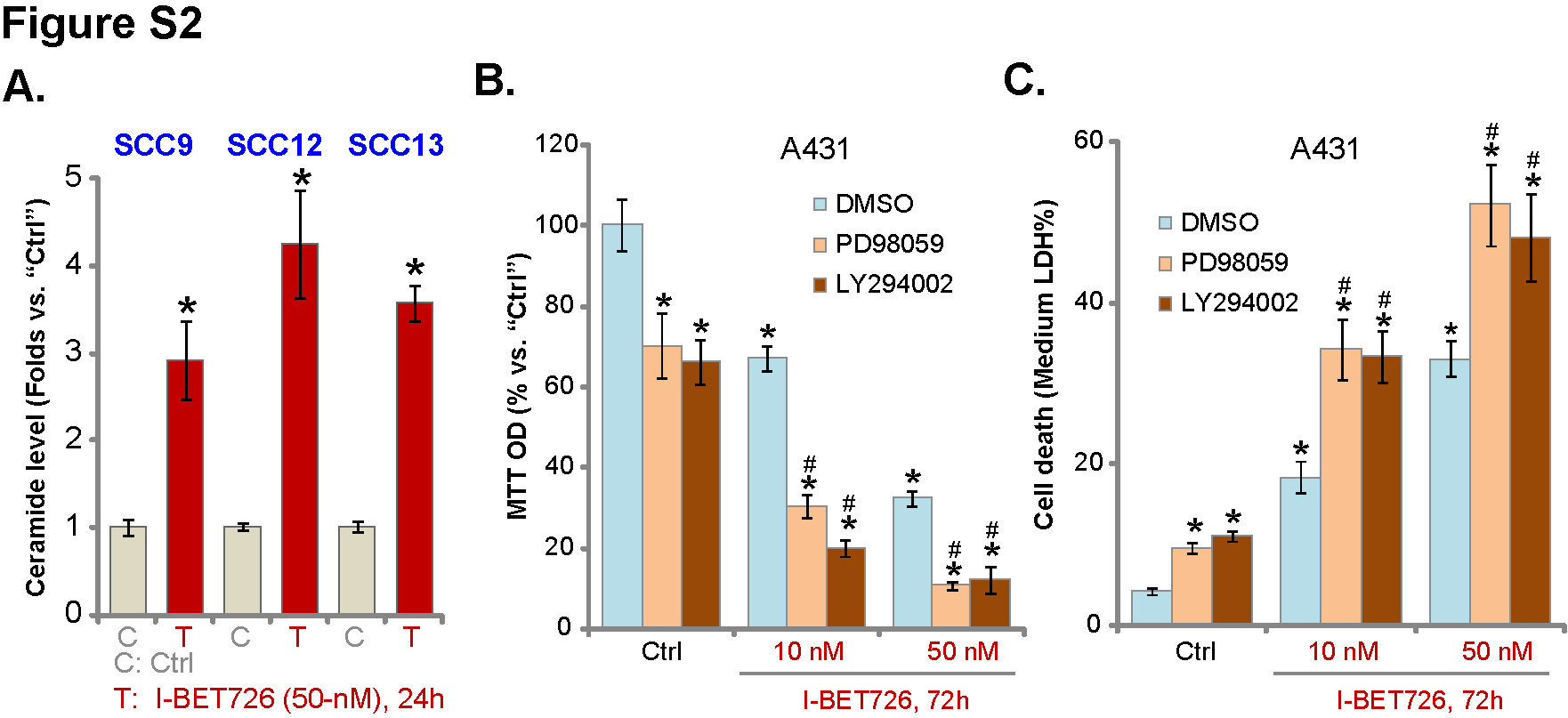

Supplement: Supplementary file 2 — Supplementary Figure 2 [file 41419_2020_2515_MOESM2_ESM.tif]
