# Supplementary material for: I-BET726 suppresses human skin squamous cell carcinoma cell growth in vitro and in vivo
Source: Cell Death Dis. 2020 May 5;11(5):318. doi: 10.1038/s41419-020-2515-z (PMC7200671; doi:10.1038/s41419-020-2515-z)
Supplement: Supplementary file 3 — Supplementary Figure Legends [file 41419_2020_2515_MOESM3_ESM.docx]

Supplementary Figure Legends

**Figure S1.** A431 cells were treated with I-BET726 (50 nM), CPI203 (500 nM), JQ1 (500 nM) or AZD5153 (“AZD”, 100 nM) for applied time periods, cell viability (MTT assay, **A**), proliferation (BrdU ELISA assay, **B**) and apoptosis (Hoechst-33342 apoptotic nuclei staining assay, **C**) were tested. **p* < 0.05 *vs.* “Ctrl” group. ^#^ *p* < 0.05 *vs.* I-BET726 treatment group.

**Figure S2.** SCC-9, SCC-12 or SCC-13 cells were treated with I-BET726 (50 nM) for 24h, cellular ceramide levels were examined (**A**). A431 cells were treated with I-BET726 (10/50 nM), with or without PD98059 (1 μM) or LY294002 (1 μM), cells were further cultured for 72h, cell viability and death were tested by MTT (**B**) and medium LDH release (**C**) assays, respectively. “DMSO” stands for vehicle control (0.1% DMSO) (**B** and **C**). **p* < 0.05 *vs.* “Ctrl” group. ^#^ *p* < 0.05 *vs.* “DMSO” group (**B** and **C**).
